# The relationship between physical activity levels and prognosis in geriatric patients diagnosed with acute coronary syndrome

**DOI:** 10.3389/fmed.2026.1747169

**Published:** 2026-05-07

**Authors:** Gokhan Taskin, Ramazan Kiyak, Muhammet Cakas, Bahadir Caglar, Evrim Duman, Ismail Emre Deniz, Ozkan Isik

**Affiliations:** 1 Department of Emergency, Faculty of Medicine, Balikesir University, Balikesir, Türkiye; 2 Emergency Service, Bingol Genc State Hospital, Bingol, Türkiye; 3 Pulmonology Clinic, Balikesir Ataturk City Hospital, Balikesir, Türkiye; 4 Department of Biostatistics, Institute of Health Sciences, Bursa Uludag University, Bursa, Türkiye; 5 Department of Coaching, Faculty of Sport Sciences, Balikesir University, Balikesir, Türkiye; 6 Sports Sciences Application and Research Center, Balikesir University, Balikesir, Türkiye

**Keywords:** acute coronary syndrome, elderly people, MACE, myocardial infarction, physical activity

## Abstract

**Purpose:**

This study aimed to investigate the relationship between Physical Activity (PA) levels and prognosis in geriatric patients diagnosed with Acute Coronary Syndrome (ACS) in the emergency department.

**Method:**

Purposive sampling was used and 207 volunteer geriatric ACS patients were included. Since the obtained data did not follow a normal distribution, the Kruskal-Wallis H test, Dunn-Bonferroni test, Fisher–Freeman–Halton test, Spearman correlation coefficient, and receiver operating characteristic curve were used in the data analysis.

**Results:**

The findings revealed that the PA levels of patients diagnosed with major adverse cardiac events (MACE) in the emergency department was lower than that of patients diagnosed with non-MACE and patients who refused treatment/were discharged and diagnosed with MACE within 30 days (*p* = 0.001). Additionally, when comparing ACS risk scores (TIMI, HEART, and SVEAT), ACS risk scores were higher in patients diagnosed with MACE compared to patients diagnosed with non-MACE and patients who refused treatment or were diagnosed with MACE within 30 days after discharge (*p* = 0.001). Patients’ PA levels were found to be negatively correlated with ACS risk scores TIMI (*r* = −0.202; *p* = 0.004), HEART (*r* = −0.387; *p* = 0.001), and SVEAT (*r* = −0.460; *p* = 0.001). ACS patients with high PA were found to have a lower MACE rate and mortality risk than patients with low and moderate PA (*p* = 0.001). Indeed, when examined patient prognosis, discharge, ward, coronary care unit, and intensive care unit services were directly related to PA (*p* = 0.001). These results indicate that higher PA may be associated with a reduced risk of ACS and mortality in geriatric individuals.

**Conclusion:**

It is recommended that PA levels be considered when making treatment decisions in geriatric ACS patients, especially in borderline cases. Furthermore, PA may be a practical and potentially useful prognostic indicator in geriatric ACS patients; however, its integration into existing ACS risk scoring systems requires further validation in larger and prospective studies.

## 1 Introduction

Cardiovascular diseases (CVD) are the leading cause of death worldwide, accounting for approximately 17.9 million deaths each year (1). Most of these deaths are due to myocardial infarction (MI) and stroke, with one-third occurring in individuals under the age of 70 (2). CVD causes high mortality and morbidity on a global scale, placing a severe burden on healthcare systems (3). In Turkey, cardiovascular mortality rates, particularly among women, are among the highest in Europe (4). These data underscores the importance of effective prevention and management strategies. One of the most critical clinical presentations within this broad spectrum of cardiovascular diseases is Acute Coronary Syndromes (ACSs). ACS is a clinical condition that develops as a result of impaired myocardial perfusion due to sudden blockage or severe narrowing of the coronary arteries. It includes Unstable Angina Pectoris (USAP), ST-elevation myocardial infarction (STEMI), and Non-ST-elevation myocardial infarction (NSTEMI) (5). MI is a component of ACS that can lead to serious consequences such as heart failure and sudden death, and contributes substantially to the global burden of CVD (6). Advanced age is an important risk factor; patients aged 75 years and older account for 35–40% of all cases, and the 8-year mortality rate in this group reaches 77% (7). The mortality risk in geriatric ACS patients is approximately twice that of younger patients (8). Therefore, ACS is a critical syndrome that requires careful monitoring, especially in older individuals.

The classic symptom of ACS is chest pain that may radiate to the neck/jaw/left arm/back. Cold sweats, dyspnea, palpitations, nausea, or syncope often accompany it. Detailed history and symptom analysis are particularly important, as they guide triage and further investigation, such as electrocardiography (ECG) and troponin testing, in the emergency department (5, 9). However, ACS often presents atypically in the geriatric population, women, and diabetics. Dyspnea, excessive fatigue, indigestion, or nausea may be present without significant chest pain (10). Atypical symptoms such as dyspnea, fatigue, and gastrointestinal complaints are more common in older adults (11). Atypical presentation leads to delays in diagnosis and misdiagnosis, reducing treatment opportunities (12). Life-saving interventions such as thrombolysis are less frequently performed in patients who do not report chest pain, and in-hospital mortality increases (13). Therefore, physicians should maintain a high index of suspicion for ACS, especially in geriatric and comorbid patients, and perform a detailed history and systematic evaluation.

Various risk scoring systems are used to predict prognosis and determine treatment strategy in patients presenting to the emergency department with suspected ACS. These scores aim to predict the short- and medium-term risk of Major Adverse Cardiac Events (MACE) by combining history, examination findings, ECG, and biochemical markers (14). Therefore, scoring systems such as TIMI, HEART, and SVEAT are widely used in the risk classification of ACS patients in the emergency department (15). However, beyond these risk scores, patients’ lifestyle factors also play an important role in prognosis, foremost among which is regular Physical Activity (PA). PA is a key determinant of cardiovascular health. A sedentary lifestyle associated with increases risk factors such as obesity, hypertension, dyslipidemia, and diabetes, posing a major risk for CVD (16). Regular moderate-to-vigorous or high-intensity PA significantly reduces the risk of MI and stroke (17). These benefits are mediated through improvements in endothelial function, lipid profile, insulin sensitivity, and inflammation, contributing to cardiovascular protection (18, 19).

PA has a positive effect on prognosis in patients who have experienced ACS. As part of secondary prevention, exercise is associated with improved quality of life and reduced risk of recurrent events (19). Patients who participate in cardiac rehabilitation programs or lead an active lifestyle show better clinical course and survival compared to sedentary individuals (20, 21). Therefore, guidelines recommend regular moderate exercise after MI, and rehabilitation programs have been associated with improved clinical outcomes, including in geriatric populations (14, 22, 23).

Recent studies have shown that pre-ACS physical activity levels are associated with clinical outcomes. Physically active patients experience milder ACS and fewer complications compared to sedentary patients. For example, Damigou et al. (24) have noted that as PA levels decrease, the rates of ischemia, heart failure, and cardiogenic shock increase, and that the risk of cardiac death is significantly lower in those who exercise regularly (25). Similarly, in Brazil, a lower risk of MACE during hospitalization has been reported in active ACS patients (26). Pitsavos et al. (27) reported that sedentary patients had a 2.5 times higher risk of complications than active patients. These findings suggest that PA may be an important factor associated with ACS prognosis.

Longitudinal studies have demonstrated the long-term benefits of PA in both primary and secondary prevention. Long-term follow-up studies have shown that sustained physical activity is associated with lower risk of recurrent cardiovascular events (27, 28). These findings confirm the protective effect of regular PA on heart health, while also highlighting the challenges of maintaining long-term exercise habits. Other longitudinal studies and meta-analyses also showed that increasing PA reduces CVD risk. For example, 1-year MACE risk ratios were lower in patients who increased their activity after ACS (29). Long-term cohorts showed that regular exercise strongly prevents coronary heart disease, with MI incidence being significantly lower in the most active groups compared to sedentary individuals (30). A large-scale prospective study reported that individuals who added moderate levels of activity to their daily lives had a significantly reduced risk of CVD over 10–15 years (31). These findings highlight the importance of public health studies examining the long-term cardiac effects of PA. However, there are still significant gaps in the literature; in particular, the effect of long-term changes in PA on cardiac prognosis has not been fully clarified (32). Therefore, long-term follow-ups in different age groups and populations will better reveal the true effects of lifestyle changes and contribute to the development of preventive strategies. This study aimed to investigate the relationship between PA and prognosis in geriatric patients diagnosed with ACS in the emergency department and to fill an important gap in the literature. Although the benefits of PA were well-documented in the general population and in younger individuals, data on geriatric ACS patients were limited. Additionally, the geriatric population is underrepresented in clinical trials, with only 10–20% of participants in existing studies being over 75 years of age (33). This situation makes it difficult to develop evidence-based recommendations specific to geriatric patients.

Although the protective effects of PA are well known, its role in the prognosis of geriatric ACS patients has not been fully clarified. Geriatric individuals have more comorbidities, different symptoms, and limited functional reserve compared to younger individuals; therefore, direct adaptation of data from the general population is not appropriate. For this reason, our study aimed to make an important contribution to the literature by focusing on geriatric individuals. Additionally, there was no comprehensive study in Turkey examining the relationship between PA levels and long-term MACE in geriatric patients diagnosed with ACS in the emergency department. Data on this subject were also limited in the international literature, and longitudinal studies were rare (34). This research aimed to fill the gap in the literature by shedding light on the prognostic importance of PA in geriatric ACS patients. This study aimed to investigate the relationship between PA and prognosis in geriatric patients diagnosed with ACS in the emergency department. Specifically, the relationship between pre-ACS PA (active/sedentary) and in-hospital complications, early-stage MACE, and mid-term mortality will be evaluated. Therefore, we aimed to offer a new perspective on risk assessment of geriatric ACS patients visiting the emergency department.

Hypotheses:

*H1*: Geriatric ACS patients with higher PA levels have a lower MACE ratio and mortality risk compared to sedentary patients.

*H2*: PA is associated with prognosis in geriatric ACS patients, independent of classic risk factors and comorbidities.

## 2 Methods

### 2.1 Research model

This study was designed a single-center pre-post-test observational study with a 30-day follow-up to determine the relationship between PA levels and the prognosis of geriatric patients who visited the Emergency Department of Balıkesir University Faculty of Medicine Application and Research Hospital between January 15, 2025, and July 15, 2025, with the ACS Clinic, in addition to their routine examinations. Observational studies aim to evaluate associations between variables within a defined population under real-life clinical conditions, and may include follow-up periods to assess outcomes over time (35, 36).

### 2.2 Patients

Patient selection for the study was conducted using a purposive sampling method. G*Power software (Version 3.1.9.2) was used to determine the sample size in this study. The sample size was calculated using G*Power software (Version 3.1.9.2). For an exploratory observational study in an unknown population, the minimum required sample size was determined to be ≥138, assuming an alpha level of 0.05 and a statistical power of 95% (1 − *β* = 0.95). The study included 212 patients over the age of 65 who visited the emergency department with new-onset chest pain. Five patients who presented to the emergency department with cardiac arrest were excluded from the study. Therefore, the study sample consisted of 207 geriatric patients with ACS.

### 2.3 Data collection tools

After the patients’ vital signs (pulse, blood pressure, body temperature, and Peripheral oxygen saturation) were recorded by the triage nurse, a 12-lead ECG was obtained and evaluated by the emergency physician. All diagnostic evaluations, including 12-lead electrocardiography and cardiac troponin measurements, were performed as part of routine clinical care using the hospital’s standard diagnostic equipment and laboratory systems. No study-specific, experimental, or proprietary diagnostic devices were used for this study. After the follow-up and treatment plan were determined, the patient’s height and weight were recorded, their body mass index was calculated, and the International Physical Activity Questionnaire, consisting of 7 questions, was administered to determine the PA performed by the patient in the last week. The PA was calculated and added to the patient’s file. Using the patient files, the medical history, the patient’s risk factors, ECG images, and initial troponin values, the patients’ CVD risk level was calculated according to the TIMI (37), HEART (38), and SVEAT (39) scores, and they were diagnosed with ACS. Additionally, all patient data were re-evaluated 30 days later through the hospital information management system and E-Pulse, and it was determined and recorded whether the patient had experienced MACE within 30 days after the initial emergency visit. Data were collected via a face-to-face survey method, and each participant responded to the questions for an average of 5–8 min.

#### 2.3.1 CVD Risk scoring

*TIMI:* The TIMI risk score initially focused on predicting 14-day mortality in patients with NSTEMI and USAP. The TIMI score typically includes factors such as age, the presence of at least three risk factors for coronary artery disease, known coronary artery disease, aspirin use in the last 7 days, recent angina, elevated cardiac markers, and ST-segment changes on an ECG. According to studies, a TIMI score of 0–2 supports discharging patients, while a score of 3–4 suggests clinical observation, and a score of 5–7 points suggests further investigation and treatment (37).
*HEART:* The HEART score, calculated using History, ECG, Age, Risk factors, and Troponin levels, is a clinical prediction tool used to assess the risk of MACE in patients visiting the emergency department with chest pain and to classify patients into low, intermediate, and high-risk groups. Each of the five components receives 0–2 points based on criteria, with a total score ranging from 0 to 10. A score between 0 and 3 indicates low risk, with a 6-week MACE risk of less than 2%. A score between 4 and 6 indicates moderate risk, with a 6-week MACE risk of 12–16%, while a score between 7 and 10 indicates high risk, with a 6-week MACE risk of 50–65%. According to the studies, a HEART score of 0–3 supports discharging patients, while a score of 4–6 suggests clinical observation, and a score of 7 points supports an early invasive strategy (38).
*SVEAT:* The SVEAT score, calculated using Symptoms, history of Vascular disease, ECG, Age, and Troponin levels, is a clinical prediction score developed to classify the risk of patients with possible ACS in the emergency department. Each factor is assigned a score ranging from −2 to +5. An SVEAT score <4 predicts low 30-day MACE risk and facilitates early discharge (39).

#### 2.3.2 International physical activity questionnaire—short form (IPAQ-SF)

The IPAQ-SF, developed by Craig et al. (40) and validated in Turkish by Öztürk (41), was used to determine individuals’ PA levels. In the IPAQ, physical activities were measured based on the criterion of being performed for at least 10 min at a time. The questionnaire asked about individuals’ vigorous PA, moderate PA, and walking times over the past 7 days. Vigorous and moderate activity and walking times were converted to METs corresponding to basal metabolic rate using the following calculations to calculate the total physical activity score (MET-min/week).

Walking score (MET-min/week) = 3.3*walking duration*walking days.

Moderate activity score (MET-min/week) = 4.0*moderate activity duration*moderate activity days.

Vigorous activity score (MET-min/week) = 8.0*vigorous activity duration*vigorous activity days.

Total Physical Activity Score (MET-min/week) = obtained by adding walking, moderate-intensity activity, and vigorous-intensity activity scores.

Classification of PA levels:

1. Low: Less than 600 MET-min/week.
2. Moderate: Between 600 and 3,000 MET-min/week.
3. High: Above 3,000 MET-min/week. Patients were classified according to their PA levels, and their ACSs were associated with them.

### 2.4 Ethical approval

This study was ethically approved by the Balıkesir University Health Sciences Non-invasive Research Ethics Committee, with decision number 2024/192. Before the study, the participants were informed about the study’s importance, and they provided informed consent.

### 2.5 Statistical analysis

The normality of continuous variables was examined using the Shapiro–Wilk test. Based on the normality test results, continuous variables in the study were expressed as median (minimum: maximum) values. The Kruskal-Wallis H test was used to compare continuous variables between study groups. The Dunn-Bonferroni post-hoc test was preferred to determine the source of the difference between groups. The Fisher–Freeman–Halton test was used to compare categorical variables between groups, with categorical variables presented as frequency and percentage values. Relationships between continuous variables were determined using the Spearman correlation coefficient. Receiver Operating Characteristics (ROC) curve analysis was performed to evaluate the discriminative ability of established ACS risk scores (TIMI, HEART, and SVEAT) in predicting MACE within the study population and to compare their diagnostic performance. For ROC analysis, patients were divided into two groups based on the occurrence of MACE within 30 days: those with MACE and those without MACE, and analyses were performed accordingly. Where meaningful cutoff values existed, the Youden-J statistic, sensitivity, specificity, positive predictive value, and negative predictive value of these cutoffs were calculated. The statistical analyses of the study were performed using the SPSS 25 (IBM Corp. Released 2017. IBM SPSS Statistics for Windows, Version 25.0. Armonk, NY: IBM Corp.) program, and statistical significance was set at *p* < 0.05 and *p* < 0.01.

## 3 Results

When patients were compared across groups, there were no statistically significant differences in age, gender, SBP, DBP, height, body weight, and BMI values among patients visiting the emergency department with suspected ACS (*p* > 0.05). However, statistically significant differences were found in patients’ pulse rate, SpO₂ (*p* = 0.021), troponin (*p* = 0.001), PA (*p* = 0.001), and ACS risk scores; TIMI (*p* = 0.004), HEART (*p* = 0.001), and SVEAT (*p* = 0.001) scores. Post-hoc analyses showed that the pulse rate and troponin levels of patients diagnosed with MACE were higher than those of other groups, while their SpO₂ levels were lower (*p* < 0.001). Furthermore, when the patients’ PA levels were examined, the PA levels of patients diagnosed with MACE was lower (*p* < 0.001). In addition, when patient groups were compared according to ACS risk scores, it was found that the ACS risk scores of patients diagnosed with MACE were significantly higher than those of other patient groups. Patients with MACE had higher troponin levels and ACS risk scores, and lower SpO₂ and PA levels compared to other groups (*p* < 0.001; Table 1).

**Table 1:** Comparison of descriptive statistics between patient groups.

Variables	Non-MACE within 30 days (*n* = 104)	MACE (*n* = 97)	Treatment refusal/MACE within 30 days after discharge (*n* = 6)	*p*
Age (years)	74 (65:90)	75 (65:91)	69 (69:72)	0.125
*Female*	48 (46.2%)	31 (32.0%)	1 (16.7%)	0.063
*Male*	56 (53.8%)	66 (68.0%)	5 (83.3%)	
SBP (mmHg)	145 (90:244)	137 (60:215)	154.50 (105:178)	0.332
DBP (mmHg)	80 (46:126)	80 (40:153)	90 (55:105)	0.736
Pulse (bpm)	82 (50:163)^b^	93 (42:181)^a^	77.50 (66:95)^ab^	0.001**
SpO_2_ (%)	96 (74:100)^a^	95 (50:100)^b^	96.50 (95:100)^ab^	0.021*
Troponin (ng/ml)	73.50 (6:526)^b^	311 (6:21133)^a^	46 (36:107)^b^	0.001**
Height (cm)	166 (155:180)	167 (152:186)	169.50 (164:172)	0.249
Weight (kg)	77 (53:92)	77 (55:100)	79.50 (69:85)	0.737
BMI (kg/m^2^)	27.8 (20.4–36.2)	27.6 (21.9–33.5)	27.75 (25.6–29.4)	0.974
PA levels (MET-min/week)	740 (0:6158)^a^	240 (0:2440)^b^	750 (380:1040)^a^	0.001**
TIMI	4 (1:6)^b^	5 (2:7)^a^	4 (3:5)^ab^	0.001**
HEART	5 (2:8)^b^	7 (3:10)^a^	5 (4:6)^b^	0.001**
SVEAT	0 (−5:8)^b^	9 (2:15)^a^	−1 (−4:3)^b^	0.001**

*p < 0.05; **p < 0.01. Data are expressed as median (minimum:maximum). ab, statistical differences between groups are represented by different letters. SBP, systolic blood pressure; DBP, diastolic blood pressure; BMI, Body Mass Index; SpO_2_, peripheral oxygen saturation.

There was a weak negative correlation between patients’ PA and TIMI scores (*r* = −0.202; *p* = 0.004). Additionally, there was a moderate negative correlation between patients’ PA with HEART (*r* = −0.387; *p* = 0.001) and SVEAT (*r* = −0.460; *p* = 0.001) scores (Table 2).

**Table 2:** Examination of the relationship between patients’ PA levels and ACS risk scores.

Variables	PA levels (MET-min/week)	
	*r*	*p*
TIMI	−0.202	0.004**
HEART	−0.387	0.001**
SVEAT	−0.460	0.001**

**p < 0.01.

When comparing patients’ MACE with PA, a statistically significant difference was found (*p* = 0.001). Non-MACE patients had higher moderate and high PA compared to other patient groups (70.7 and 100%, respectively). This result suggests that higher PA is associated with a lower risk of MACE in ACS patients (Table 3).

**Table 3:** Comparison of patients’ MACEs and PA levels.

Variables		MACE					
		Non-MACE	NSTEMI angiography	STEMI angiography	Elective angiography	USAP	*p*
PA (MET min/week)	Low	33 (30.3%)	31 (28.4%)	14 (12.8%)	1 (0.9%)	30 (27.5%)	0.001**
	Moderate	65 (70.7%)	5 (5.4%)	7 (7.6%)	5 (5.4%)	10 (10.9%)	
	High	6 (100%)	0 (0%)	0 (0%)	0 (0%)	0 (0%)	

**p < 0.01; Data are expressed as *n* and (%).

When compared PA based on treatment outcomes for patients admitted to the ACS clinic and the emergency department, a statistically significant difference was found (*p* = 0.001). The PA levels of discharged patients [780(0:6158) MET-min/week]; intensive care unit [130(0:1240) MET-min/week], coronary care unit [360(0:2370) MET-min/week], and the service [420(0:2440) MET-min/week] were found to be higher than the scores of patients in the intensive care unit [780(0:6158) MET-min/week]. PA levels were lower in patients requiring intensive care compared to those who were discharged (*p* = 0.001; Table 4).

**Table 4:** Comparison of PA among patients visiting the emergency department with ACS based on treatment outcomes.

Variables	PA levels (MET-min/week)
Discharged	780 (0:6158)^a^
Service	420 (0:2440)^b^
Coronary care unit	360 (0:2370)^b^
Intensive care unit	130 (0:1240)^b^
*p*	0.001**

**p < 0.01. Data are expressed as median (minimum:maximum). ab, statistical differences between groups are represented by different letters.

**Figure 1:**
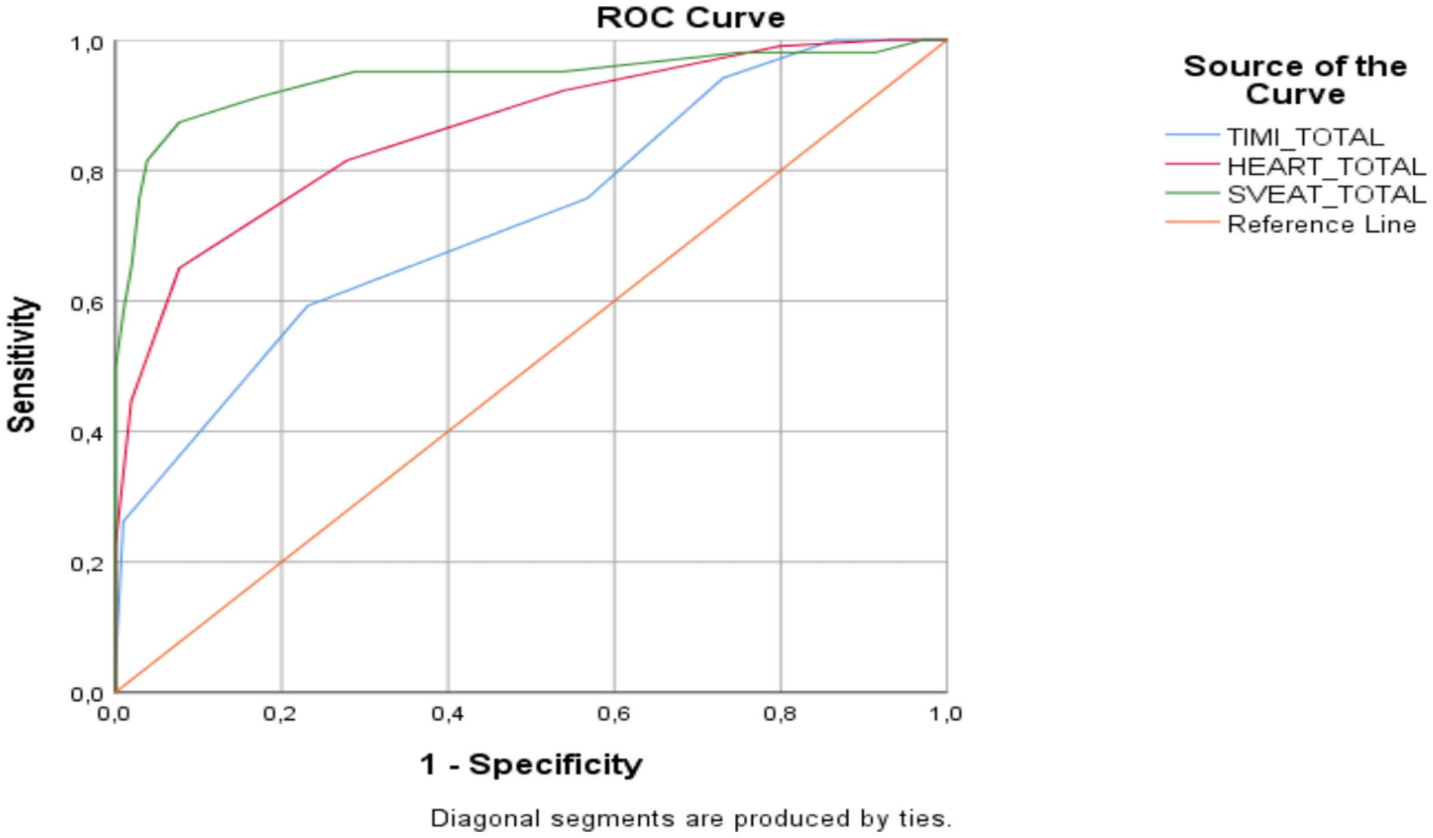
ROC curve for ACS risk scores.

When patients were classified according to ACS risk scores cutoff points, the areas under and above the ROC curves were significantly different (*p* = 0.001; Figure 1). This result indicates that ACS risk scores can distinguish between two diagnoses (Non-MACE and MACE). For each ACS risk score, the cutoff points indicate the highest levels of sensitivity and specificity (*p* = 0.001; Table 5).

**Table 5:** ROC analysis for ACS risk scores.

Variables	95% CI							
	Youden J	AUC	Cutoff	Specificity	Sensitivity	Lower bound	Upper bound	*p*
TIMI	0.3615	0.727	>4	76.9	59.2	0.661	0.787	0.001**
HEART	0.5736	0.861	>6	92.3	65	0.807	0.905	0.001**
SVEAT	0.7969	0.940	>3	92.3	87.4	0.898	0.968	0.001**

**p < 0.01; positive group = 103 people; negative group = 104 people.

## 4 Discussion and conclusion

In this study, the role of PA in the short-term prognosis of geriatric ACS patients was investigated and evaluated in comparison with current literature. In our study, troponin levels and ACS risk scores were found to be significantly higher in patients diagnosed with MACE compared to those diagnosed with non-MACE and those who refused treatment and/or were diagnosed with MACE within 30 days after discharge (Table 1). Additionally, a negative correlation was found between ACS risk scores and PA of geriatric patients. This result indicates that as PA levels increases, ACS risk scores decrease (Table 2). Furthermore, it was found that moderate PA was associated with a lower proportion of MACE, and no MACE events were observed in geriatric patients with high PA levels diagnosed with ACS in the ED (Table 3). This result suggests a potential protective association between PA and clinical prognosis. Similar findings exist in the literature; for example, Pitsavos et al. (27) reported a significant reduction in the risk of cardiovascular events in patients who performed regular PA, while Cho et al. (42) reported a marked decrease in the incidence of MACE in patients who increased their PA after ACS. Our study adapts these data to the geriatric population and demonstrates that high PA level can reduce the risk of MACE. This finding reveals that PA is a modifiable factor that not only improves quality of life but also prolongs life, particularly in the geriatric population. Although our findings suggest a significant association between PA level and clinical outcomes, the predictive value of PA level as an independent prognostic marker was not formally evaluated using multivariable models. Therefore, future studies are needed to assess whether PA level provides incremental prognostic value beyond established risk scores.

At this point, the results of our study, together with other results in the literature, suggest that PA can be considered a clinically relevant parameter in clinical practice. A recent prospective study reported that regular PA during the pre-MI period significantly reduced 1-year mortality (27). The findings of our study are consistent with these results. So, routine PA level assessment in the emergency department may be recommended. As seen in our study, PA may provide additional prognostic information beyond established ACS risk scores. This highlights the importance of considering PA levels of geriatric patients, especially in borderline cases, when making treatment decisions. In conclusion, PA may be considered a potentially useful prognostic marker that is easily measurable, modifiable, and can be integrated into ACS risk scoring systems.

The use of PA level as a prognostic indicator is an increasingly important topic, especially in geriatric heart patients (43). Our findings show that simply knowing how active a patient is in daily life can be valuable in predicting the course after ACS. Our finding of low mortality and MACE ratio in patients with high PA suggests that PA may be an independent prognostic indicator (Table 3). Reviewing the literature, Pitsavos et al. (27) reported that in a large population of 2,172 patients with ACS who performed regular PA, troponin levels were lower at hospital admission, in-hospital mortality was reduced by 44%, and the MACE risk at 30 days was 20% lower. Similarly, a recent study reported that patients who had undergone MI and performed ≥150 min of aerobic exercise per week had a significantly reduced 1-year MACE risk and approximately 50% lower CVD-related mortality (44). The prognostic effect of regular PA is also seen in cardiac rehabilitation studies; for example, a meta-analysis study showed that exercise-based cardiac rehabilitation in ACS patients reduced the risk of MACE by 84% (21). These data indicate that the findings of our study are consistent with the current literature, suggesting that PA may play a critical role in reducing the risk of MACE. Our results suggest that PA in geriatric ACS patients may be an important indicator in clinical risk assessment. Clinically, these results suggest that regular PA may increase tolerance to myocardial ischemia and reduce MI size by developing collateral circulation. This is consistent with findings in the literature suggesting that an active lifestyle may reduce the severity of ACS (26). Clinically, physicians may consider PA history in ACS patients as a prognostic indicator, and a sedentary lifestyle may be thought to increase ACS risk in geriatric patients.

Indeed, the results of our study showed that the PA of patients discharged from the ACS clinic based on treatment outcomes were statistically significantly different from those of patients admitted to the emergency department, coronary care unit, and/or intensive care unit. This result indicates that achieving a moderate/high PA level in geriatric patients has a protective effect against the risk of ACS (Table 4). In light of these findings, it can be said that PA level should be routinely assessed as a “vital sign” in clinical evaluations of ACS patients. Obtaining the patient’s PA history through simple questions (e.g., how much they walk daily, whether they exercise) or short questionnaires such as the IPAQ-SF will contribute to a clearer risk profile when combined with other clinical and laboratory data. In our study, the prognostic value of PA provided an additional contribution independent of current ACS risk scores. This highlights the importance of considering the patient’s physical performance, especially in borderline cases, when making treatment decisions. In conclusion, PA can be considered an easily measurable and modifiable prognostic marker that can be integrated into ACS risk scoring systems. The results in Table 4 of our study showed that patients with high PA level were discharged more frequently, while those with low activity levels had significantly higher rates of intensive care or service admission. This situation reveals that PA is closely related not only to MACE but also to the severity of the in-hospital treatment process. Similarly, the ROC analysis in Table 5 shows that the SVEAT score has higher discriminatory power compared to other risk scores, suggesting that this score can be used more reliably in clinical practice in geriatric patients. However, while the absence of any MACE in patients with high PA is noteworthy given the sample size, it should be noted that this relationship is limited for absolute generalization. Furthermore, the lower performance of the TIMI and HEART scores compared to SVEAT indicates that these scores do not adequately reflect functional capacity and frailty in the geriatric population. Therefore, although our findings are generally consistent with the current literature, they require confirmation with larger samples and more prospective studies.

Studies evaluating the relationship between parameters such as PA history and short- and long-term outcomes in patients diagnosed with ACS in the emergency department are rare (25). In this regard, our study fills an important gap by demonstrating the prognostic importance of PA level in a group of geriatric patients with comorbidities under real-life conditions. Additionally, it is the first to highlight the relationship between PA and common risk scores such as TIMI, HEART, and SVEAT. The literature suggests that the newly developed SVEAT score may be superior to the HEART score, but neither score accounts for functional status in geriatric patients (45). Our study demonstrates that PA level provides an independent benefit from these scores, offering insights into how they could be improved in the future. Furthermore, our research supports the emphasis that participation in secondary prevention strategies (e.g., cardiac rehabilitation) is generally low in geriatric patients (46). This is another point that makes our study unique, and our findings shed light on this neglected aspect of geriatric patients. In conclusion, although the discriminative performance of physical activity as a standalone predictor was not evaluated using ROC analysis in the present study, PA can still be considered a potentially useful prognostic marker that is easily measurable, modifiable, and may be integrated into ACS risk scoring systems.

Considering our findings, we recommend routinely assessing PA in the acute evaluation and follow-up of ACS patients. Particularly in geriatric patients, it should be quickly assessed whether they are physically active and the extent to which they can maintain their daily living activities. In this way, patients who lead a sedentary lifestyle and are likely to be more fragile can be identified early and placed under closer monitoring or aggressive risk factor control. Although lifestyle changes are recommended for all ACS patients after hospital discharge for secondary prevention, this should be particularly emphasized in geriatric patients. Physicians should be proactive in referring older patients to personalized exercise plans and cardiac rehabilitation programs (33). As geriatric patients participate less in secondary prevention programs due to reduced functional capacity and multiple comorbidities. However, appropriately individualized PA programs can provide physical, cognitive, and psychological benefits in these patients (47). Invasive procedure decisions should also consider not only the patient’s age but also their physical capacity and overall condition. For example, an 80-year-old ACS patient who is still active in daily life may be considered biologically younger and thus have a high potential to benefit from invasive interventions. In contrast, a patient of the same age but with severe functional limitations may require a more conservative approach and palliative care plans (33). Finally, cardiac rehabilitation opportunities should be made available to geriatric patients. This allows geriatric patients with low PA and high risk to participate safely in exercise, improving their prognosis in the medium to long term.

Another finding of our study was that patients with high PA were discharged more frequently, while those with low PA had significantly higher rates of intensive care unit or service admission. This finding demonstrates that PA is closely associated not only with MACE but also with the severity of the in-hospital treatment process. Similarly, the ROC analysis in Table 5 shows that the SVEAT score has higher discriminatory power compared to other risk scores, suggesting that the SVEAT score may be more reliably used in clinical practice than TIMI and HEART in geriatric patients. However, while the absence of any MACE in patients with high PA is noteworthy given the sample size, it should be noted that this relationship is limited for absolute generalization. Furthermore, the lower performance of the TIMI and HEART scores compared to SVEAT suggests that these scores may not adequately reflect functional capacity and frailty in the geriatric population. Therefore, while these findings are generally consistent with literature, they need to be confirmed with larger samples and more prospective studies.

This study has several limitations. First, the single center pre-post-test observational design limits the ability to establish causal relationships between PA and clinical outcomes. Second, PA were assessed using a self-reported questionnaire, which may be subject to recall bias and may lead to over- or underestimation of actual activity levels. Third, the study was conducted at a single center, which may limit the generalizability of the results to other populations. Another limitation of the study is the relatively small number of patients in the subgroup who refused treatment and developed MACE within 30 days. This imbalance in group sizes may have affected the statistical power and reliability of comparisons involving this subgroup, and the results should therefore be interpreted with caution. Additionally, PA was evaluated based on pre-admission data, and changes in activity patterns after discharge were not assessed. Furthermore, potential confounding variables were not controlled using multivariable analysis, which should be considered when interpreting the findings. Finally, although the sample size was adequate for the study design, larger and multicenter studies are needed to confirm these results.

### 4.1 Suggestions

In the future, prospective, larger sample studies should be conducted to better understand the prognostic impact of PA in geriatric ACS patients. Studies examining the effect of pre-disease PA level on long-term cardiac outcomes would be particularly valuable. Interventional studies may also be planned. For example, randomized controlled trials evaluating the effect of structured exercise programs on MACE and survival in sedentary geriatric ACS patients could be designed. This would clarify the causal effect of PA and the extent to which it is a modifiable risk factor. Another area of research is the integration of physical performance measures into current ACS risk scores. For example, it could be investigated whether a new ACS risk scoring system could be developed that combines measurements such as a simple walking speed test or a sit-to-stand test. With advances in technology, it is also possible to objectively monitor the activity levels of geriatric patients using wearable devices; the prognostic effects of changes in PA in the post-ACS period can be investigated using these devices. Since geriatric patients are generally underrepresented in research, it is difficult to generate evidence specific to their needs (48). Researchers can fill this gap by designing study protocols for the older age group and, if necessary, using flexible intervention models (e.g., home-visit rehabilitation, telehealth applications). The common goal of all these recommendations is to best assess the decisive impact of PA on survival and quality of life in geriatric ACS patients and to develop care standards based on this information.

## Data Availability

The raw data supporting the conclusions of this article will be made available by the authors, without undue reservation.
